# *Mlh1* heterozygosity and promoter methylation associates with microsatellite instability in mouse sperm

**DOI:** 10.1093/mutage/geab010

**Published:** 2021-03-19

**Authors:** Kul S Shrestha, Minna M Tuominen, Liisa Kauppi

**Affiliations:** 1 Systems Oncology (ONCOSYS) Research Program, Research Programs Unit, Faculty of Medicine, University of Helsinki, Haartmaninkatu 8 (PO Box 63), FI-00014 Helsinki, Finland; 2 Doctoral Program in Integrative Life Sciences, University of Helsinki, Viikinkaari 1 (PO Box 65), FI-00014 Helsinki, Finland; 3 Department of Biochemistry and Developmental Biology, Faculty of Medicine, University of Helsinki, Haartmaninkatu 8 (PO Box 63), FI-00014 Helsinki, Finland

## Abstract

DNA mismatch repair (MMR) proteins play an important role in maintaining genome stability, both in somatic and in germline cells. Loss of MLH1, a central MMR protein, leads to infertility and to microsatellite instability (MSI) in spermatocytes, however, the effect of *Mlh1* heterozygosity on germline genome stability remains unexplored. To test the effect of *Mlh1* heterozygosity on MSI in mature sperm, we combined mouse genetics with single-molecule PCR that detects allelic changes at unstable microsatellites. We discovered 4.5% and 5.9% MSI in sperm of 4- and 12-month-old *Mlh1*^+/−^ mice, respectively, and that *Mlh1* promoter methylation in *Mlh1*^+/−^ sperm correlated with higher MSI. No such elevated MSI was seen in non-proliferating somatic cells. Additionally, we show contrasting dynamics of deletions versus insertions at unstable microsatellites (mononucleotide repeats) in sperm.

## Introduction

DNA mismatch repair (MMR) plays a crucial role in maintaining post-replicative genomic stability. During spermatogenesis, in pre-meiotic cells, MMR proteins MLH1, PMS2, MSH2, MSH3 and MSH6 are involved in repairing insertion–deletion (indel) mutations and single base pair mismatches, and in meiotic cells, MMR proteins MLH1, MLH3, MSH4 and MSH5 are essential for ensuring meiotic crossovers (1–5). Loss-of-function of any of these genes leads to adverse consequences in genomic stability leading to various abnormalities or even infertility (1,3,6–10).

Spermatogenesis involves high levels of cell proliferation. Spermatogonial stem cells either self-renew or undergo 9–11 mitotic divisions to produce spermatocytes, which subsequently undergo meiotic cell divisions to produce haploid sperm cells (male gametes) (11). Hence, a mature sperm cell is a product of numerous rounds of DNA replication.

DNA replication is inherently mutagenic. In eukaryotes, during each round of replication, DNA polymerases α, δ and ε make on average less than 1 × 10^−5^ replication errors per nucleotide (12–14). The intrinsic proofreading activity of DNA polymerase corrects most of these errors, and post-replication, nearly all remaining errors are repaired by MMR. An average of 1.8 × 10^−10^ mutations per nucleotide is introduced into the mouse genome during every cell division (15). Short tandem repeat sequences in the genome called microsatellites are particularly prone to replication errors, by a process known as polymerase ‘slippage’. DNA polymerase often erroneously inserts or bypasses individual repeat units at microsatellites, resulting in small indel loops between the parental DNA strand and the newly replicated daughter strand. If left unrepaired, indel loops give rise to mutant alleles of novel microsatellite repeat array lengths. This molecular phenotype is known as microsatellite instability (MSI). During spermatogenesis, several rounds of DNA replication take place before cells enter meiosis. It follows that any MSI detected in sperm is likely pre-meiotic, i.e. it originates in spermatogonia which is the only cell type in the testis to undergo extensive cell proliferation.

MMR is crucial for microsatellite stability in both somatic and germline tissues (6,7,9,16). Individuals with inherited MMR defects, in particular *MLH1* and *MSH2* heterozygous mutations, a condition known as Lynch syndrome (LS) often develop MSI-associated colorectal cancer, endometrial cancer and various other cancers once the single functional MMR allele is lost (7,17–20). Germline and/or sporadic promoter methylation of MMR genes also leads to MSI-associated cancers (21,22). Further, MMR defects severely affect fertility and germline MSI in humans (3,23,24) and in mice (1,9). Both male and female *Mlh1*^−/−^ mice are infertile, and male *Mlh1*^−/−^ mice exhibit spermatocyte MSI (1,9). Despite the severity of germline phenotypes in *Mlh1*^−/−^ mice, there is very limited knowledge on how heterozygosity of *Mlh1* (i.e. *Mlh1*^+/−^) impacts MSI in germline cells.

Here, we investigate how *Mlh1* heterozygosity affects MSI in sperm cells, and also assess spleen MSI to obtain a germline versus somatic MSI comparison. Further, by assaying *Mlh1* promoter methylation status in *Mlh1*^+/−^ sperm, we establish a correlation between MSI and *Mlh1* promoter methylation in the germline. In addition, we establish estimates of the contribution of insertions and deletions to sperm MSI.

## Materials and methods

### Mice, genotyping and tissue collection

The mice used in this study were *Mlh1* mice (B6.129-*Mlh1*^tm1Rak^, strain 01XA2, National Institutes of Health, Mouse Repository, NCI Frederick) (9). National and institutional guidelines (Animal Experiment Board in Finland and Laboratory Animal Centre of the University of Helsinki) were followed throughout. *Mlh1* genotyping was performed using genomic DNA extracted from earpieces (see Supplementary information for the genotyping protocol). Tissues were collected from 4- and 12-month-old mice, snap-frozen and stored at −80°C until further use. Three *Mlh1*^+/+^ and six *Mlh1*^+/−^ mice per age group were used in this study.

### DNA extraction from sperm cells and spleen

Mature sperm were isolated from cauda epididymides of 4- and 12-month-old mice according to a previously published protocol (25), briefly as follows. For each mouse, cauda epididymides were finely chopped using a razor blade. The chopped pieces were transferred into a microcentrifuge tube containing 1× saline-sodium citrate (SSC; 0.15 mM NaCl, 15 mM sodium citrate) and incubated for 20 min at room temperature. Then the cell suspension was repeatedly pipetted up and down for 2 min to release the sperm cells. Cells were washed twice with 1× SSC. Somatic cells were lysed with 0.15% (w/v) sodium dodecyl sulphate (SDS). Sperm cell purity was assessed under a microscope by counting the number of sperm cells versus non-sperm cells using 5 µl of the cell suspension. We obtained sperm cell purity of over 95%. Sperm cells were washed twice with 0.2× SSC and centrifuged for 3 min at full speed. The supernatant was removed, the sperm pellet was resuspended into 300 µl of buffer containing 100 mM Tris-HCl (pH 8.0), 10 mM EDTA, 500 mM NaCl, 1% SDS and 1 M β-mercaptoethanol supplemented with 100 µl of 20 mg/ml proteinase K, and incubated overnight at 56°C to lyse the sperm heads. The next day, sperm DNA was extracted using DNeasy Blood & Tissue Kit (Qiagen, Hilden, Germany) according to the manufacturer’s instruction. The same kit [DNeasy Blood & Tissue Kit (Qiagen)] was used to extract DNA from the spleen of 4- and 12-month-old mice. Briefly, ~2 mg of tissue was finely chopped with a surgical blade and transferred to the kit’s lysis buffer. Further homogenisation was done using a 20G needle and syringe. Thereafter, column-based DNA extraction was performed according to the manufacturer’s instruction.

### Single-molecule MSI analysis by PCR

Extracted DNA was quantified using a Qubit fluorometer (Thermo Fisher Scientific, Waltham, MA, USA) and diluted to a concentration of approximately five DNA molecules/µl (assuming 3 pg DNA per haploid mouse genome) in 5 mM Tris-HCl (pH 7.5) supplemented with 5 ng/µl carrier (sheared) herring sperm DNA (Thermo Fisher Scientific). MSI was assayed at single-DNA molecule level using single-molecule PCR (SM-PCR) (26–28). Three microsatellites were tested for MSI: two mononucleotide repeat loci A27 and A33 (29), and one dinucleotide repeat locus D14Mit15 (9). Mononucleotide tract A27 is an intergenic microsatellite located ~2 kb downstream of the *Epas1* gene, A33 resides within the *Epas1* gene (between exons 2 and 3), and D14Mit15 is an intergenic microsatellite at 40 kb distance from the *Ptpn20* gene.

PCR was performed using the Q5 High-Fidelity DNA Polymerase system (New England Biolabs, Ipswich, MA, USA), supplemented with 1 ng/µl carrier (sheared) herring sperm DNA in a 10 µl reaction volume. To ensure that individual PCRs in SM-PCR are seeded with a single amplifiable DNA molecule, for each DNA sample to be analysed we determined, using a dilution series, the DNA concentration that yielded 50% PCR success rate similar to previous reports (27,30). This DNA concentration was determined separately for each of the three microsatellite loci assayed. By Poisson approximation, 50% PCR success rate equates to approximately one amplifiable molecule per positive reaction (27,30–32). Subsequent MSI analysis was run in 96-well PCR plates. Each PCR was seeded with approximately one amplifiable DNA molecule, and each PCR plate included four wells of PCR mix only (no template DNA added) as negative controls. Previously published primers (8,29) were used for PCR as follows: for A27, 0.5 µM each of primer (A27_F 5′-6-FAM-TCCCTGTATAACCCTGGCTGACT-3′ and A27_R 5′-GCAACCAGTTGTCCTGGCGTGGA-3′), for A33, 0.2 µM each of primer (A33_F 5′-VIC-TACAGAGGATTGTCCTCTTGGAG-3′ and A33_R 5′-GCTGCTTCACTTGGACATTGGCT-3′), and for D14Mit15, 0.1 µM of each primer (D14Mit15_F 5′-NED TTGGCTGCTCACTTGCAG-3′ and D14Mit15_R 5′-TTACCCTCCCCATAACTCCC-3′). A33 and D14Mit15 were assayed in the same PCR, and a separate PCR was run for A27. For A33 and D14Mit15 duplexed PCR, the following PCR program was used: 30 s at 98°C, 35 cycles of 10 s denaturation at 98°C, 30 s primer annealing at 66°C, 5 s extension at 72°C, followed by 2 min final extension at 72°C. For A27, the aforementioned PCR program was used, except for the primer annealing temperature being 70°C. 1 µl of each PCR product was used for fragment analysis. Fragment analysis was performed by capillary electrophoresis, with an internal size standard (GeneScan™ 500 LIZ™ dye Size Standard, Applied Biosystems, Waltham, MA, USA), using ABI3730xl DNA Analyzer (Thermo Fisher Scientific). Between 121 and 259 amplifiable DNA molecules per sample were assayed for each microsatellite locus. Data were analysed using the Fragman R package (33). Stringent criteria were used for true microsatellite signal calling and for mutant scoring [adopted from (27,34)], and thus the mutation rates reported here are likely a conservative estimate. The criteria were as follows:

1. A true microsatellite signal should have lower-intensity stutter peaks. Stutter peaks should display the expected size difference (i.e. 1 base for mononucleotide repeats and 2 bases for dinucleotide repeats) from the dominant peak. Reactions with peaks without stutter were considered artefacts.
2. For an allele to be considered as mutant, both the highest peak and the stutter peaks should shift as a single unit. Shift of the highest peak alone was not scored as a mutant.
3. If a wild-type and (apparently) mutant allele co-occurred in a single PCR, the reaction was scored as wild type. Non-wild-type peaks were presumed to result from replication slippage during the early rounds of PCR and thus considered artefacts.

For each of the three microsatellite loci assayed, MSI was separately scored for insertions and deletions. MSI rate was calculated as follows:


$$\begin{array}{ll} MSI\%\;= & (total\;no.\;of\;single\;repeat\;unit\;shifts\;observed/\\ & total\;DNA\;molecules\;analyzed)\;\times \;100\% \end{array}$$

### *Mlh1* promoter methylation analysis by methylation-specific PCR (MSP)

Methylation status of the *Mlh1* promoter in sperm cells and splenic cells was tested using MSP assay (35). The same DNA (undiluted stock DNA) as used in the MSI assay was used for MSP. For MSP, 200 ng of DNA was bisulphite-converted using EZ DNA Methylation-Direct Kit (Zymo Research, Irvine, CA, USA) according to manufacturer’s instruction, and 1 µl of bisulphite-converted DNA was used for PCR. PCR was performed in 2× Zymo *Taq* premix system (Zymo Research) with previously published primers (36). Two separate PCRs, one with a primer pair targeting methylated *Mlh1* promoter (0.8 µM each of forward primer 5′-GAATTTGAGCGTGAGGAGTTC-3′ and reverse primer 5′-TAACCGACCGCTAAATAACTTCC-3′), and the other with primer pair targeting unmethylated *Mlh1* promoter (0.8 µM each of forward primer 5′-AGAATTTGAGTGTGAGGAGTTT-3′ and reverse primer 5′-CCAACCACTAAATAACTTCCC-3′) were performed using the following PCR program: 10 s at 95°C, 40 cycles of 30 s at 95°C, 30 s at 62°C and 60 s at 72°C, and final extension for 7 min at 72°C. Universally methylated mouse DNA standard (cat. no. D5012, Zymo Research) and *Mlh1*^+/+^ spleen DNA (from 1-month-old mouse) were used as positive and negative controls, respectively. The PCR products were analysed on a 1.5% agarose gel in the presence of ethidium bromide and visualised with UV light. Methylation status of the *Mlh1* promoter was scored qualitatively based on the presence or absence of the 143-bp amplification product after PCR with primer pair specific to methylated CpG site.

### Statistical analysis

Unpaired *t*-test was used to test the differences in MSI rates between the groups. Two-tailed *P* values <0.05 were considered to be statistically significant.

## Results

### *Mlh1* ^+/−^ sperm cells display MSI at mononucleotide repeats

**Fig. 1.**
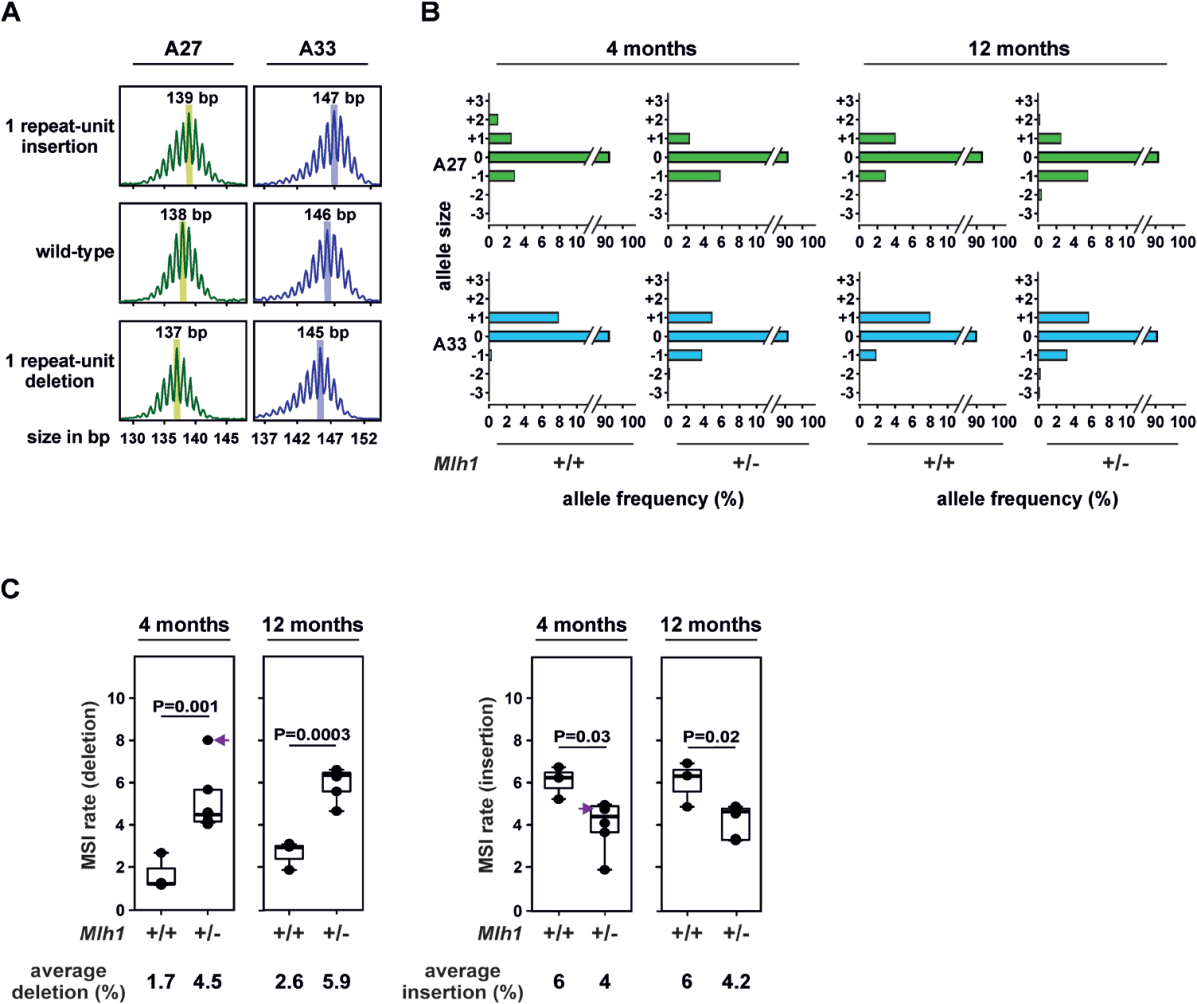
Sperm MSI at mononucleotide repeats. (**A**) Representative capillary electropherograms of most commonly observed alleles at A27 and A33. Shown are a single repeat unit insertion (top panel), the wild-type allele (middle panel) and a single repeat unit deletion (bottom panel). Highlighted with the shaded rectangles are the allelic peaks which were scored; smaller peaks flanking this highest peak are stutter peaks (a typical PCR artefact for microsatellites). (**B**) Various alleles observed at mononucleotide repeat markers A27 and A33, expressed as percentage of amplifiable DNA molecules assayed. On the *y*-axis, ‘+’ indicates gains, i.e. insertions, and ‘-’ indicates losses, i.e. deletions of repeat units. (**C**) Mononucleotide repeats display more deletions and fewer insertions in *Mlh1*^+/−^ sperm compared to age-matched *Mlh1*^+/+^ sperm. The data points in boxplot represent weighted average of MSI at A27 and A33. Indicated are the *P* values for wild-type and *Mlh1*^+/−^ sperm MSI rate comparisons using unpaired *t*-test. Arrow indicates the outlier *Mlh1*^+/−^ mouse. *n* = 3 and 6 for *Mlh1*^+/+^ mice and *Mlh1*^+/−^ mice, respectively, for each time point.

We assayed sperm MSI by SM-PCR to investigate the effects of *Mlh1* heterozygosity on germline microsatellite stability. We assessed sperm DNA of 4- and 12-month-old *Mlh1*^+/−^ mice. Sperm DNA from age-matched *Mlh1*^+/+^ littermates (or from closely related matings) was used as controls. We tested MSI at three microsatellites: two mononucleotide repeats A27 and A33 (Figure 1A) and one dinucleotide repeat D14Mit15 (Supplementary Figures 1 and 2). MSI was scored separately for insertions and deletions.

While the dinucleotide D14Mit15 repeat was stable (Supplementary Figure 1), both mononucleotide repeats displayed MSI in *Mlh1*^+/−^ sperm. *Mlh1*^+/−^ sperm showed substantially more 1-bp deletions than age-matched wild-type sperm (which also showed low levels of 1-bp deletions), at both 4- and 12-month time points (Figure 1B). Both *Mlh1*^+/+^ and *Mlh1*^+/−^ sperm showed an increase in deletions with age (Figure 1C). This increase was significant (*P* = 0.008) in *Mlh1*^+/−^ sperm, but not in *Mlh1*^+/+^ sperm. Compared to age-matched *Mlh1*^+/+^ sperm, *Mlh1*^+/−^ sperm had significantly more deletions (*P* = 0.001 and *P* = 0.0003 for 4- and 12-month time points, respectively), with 2.7- and 2.3-fold higher deletion rates at 4- and 12-month time points, respectively (Figure 1C). One 4-month-old *Mlh1*^+/−^ mouse (indicated by an arrow in Figure 1C) showed higher deletions (8%) in sperm compared to other *Mlh1*^+/−^ mice in the same age group. By using Grubbs’ test, this mouse was categorised as an outlier (*P* < 0.05) and was omitted from statistical analyses.

In *Mlh1*^+/+^ sperm, insertions were more common than deletions at both time points (*P* = 0.002 for insertions versus deletions comparison for both 4- and 12-month time points). Compared to age-matched *Mlh1*^+/+^ mice, sperm from *Mlh1*^+/−^ mice showed fewer insertions at both time points (Figure 1C). Insertions were predominantly single repeat unit (i.e. 1 bp) in size (Figure 1B), and there was no considerable change in insertion% with age in *Mlh1*^+/+^ or *Mlh1*^+/−^ sperm (Figure 1C).

### *Mlh1* promoter methylation is frequent in sperm of *Mlh1*^+/−^ mice and associates with MSI

**Fig. 2.**
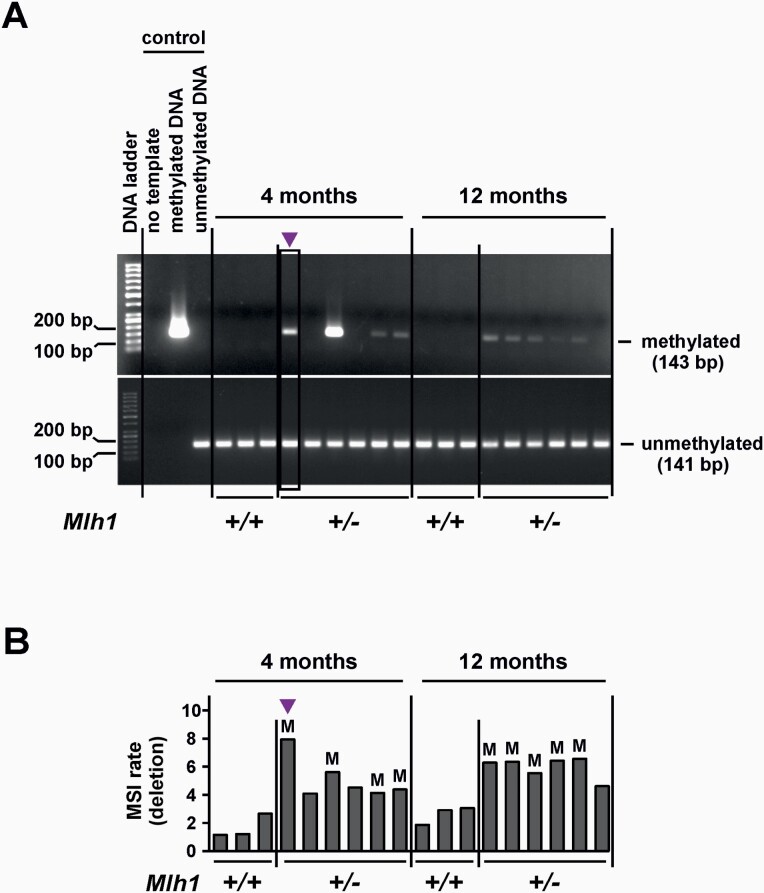
*Mlh1* promoter methylation in sperm correlates with germline MSI. (**A**) Representative gel image of methylation-specific PCR (MSP) for *Mlh1* promoter. Upper and lower gel images show products for MSP-PCRs using primers specific to methylated and unmethylated *Mlh1* promoter, respectively. (**B**) Deletions (percentage of total molecules assayed) in *Mlh1*^+/−^ sperm (same data as in Figure 1C), with *Mlh1* promoter methylation status indicated for each sperm sample with ‘M’. Samples are in the same order as in gel image above (A). Arrow indicates the outlier *Mlh1*^+/−^ sperm sample.

We used MSP to test *Mlh1* promoter methylation status in *Mlh1*^+/−^ sperm, and to investigate whether germline *Mlh1* promoter methylation correlates with germline MSI. The *Mlh1* promoter in a given sample was scored as methylated if an amplification product (143 bp in size) was detected by PCR with methylation-specific primers. A representative gel image of the MSP assay is shown in Figure 2A.

None of the *Mlh1*^+/+^ mice assayed showed *Mlh1* promoter methylation in sperm (Figure 2A). In *Mlh1*^+/−^ mice, *Mlh1* promoter methylation was detected in 67% (4 out of 6) and 83% (5 out of 6) sperm DNA samples at 4- and 12-month time points, respectively (Figure 2A). *Mlh1* promoter methylation was associated with elevated deletions (Figure 2B), but not insertions (Supplementary Figure 3) in sperm.

MSP was also performed in spleen. All *Mlh1*^+/−^ mice with *Mlh1* promoter methylation in sperm displayed *Mlh1* promoter methylation in spleen, while those without promoter methylation in sperm did not (Supplementary Figure 4). *Mlh1* promoter methylation was not observed in spleen of *Mlh1*^+/+^ mice.

### MSI is higher in *Mlh1*^+/−^ sperm than in *Mlh1*^+/−^ spleen

**Fig. 3.**
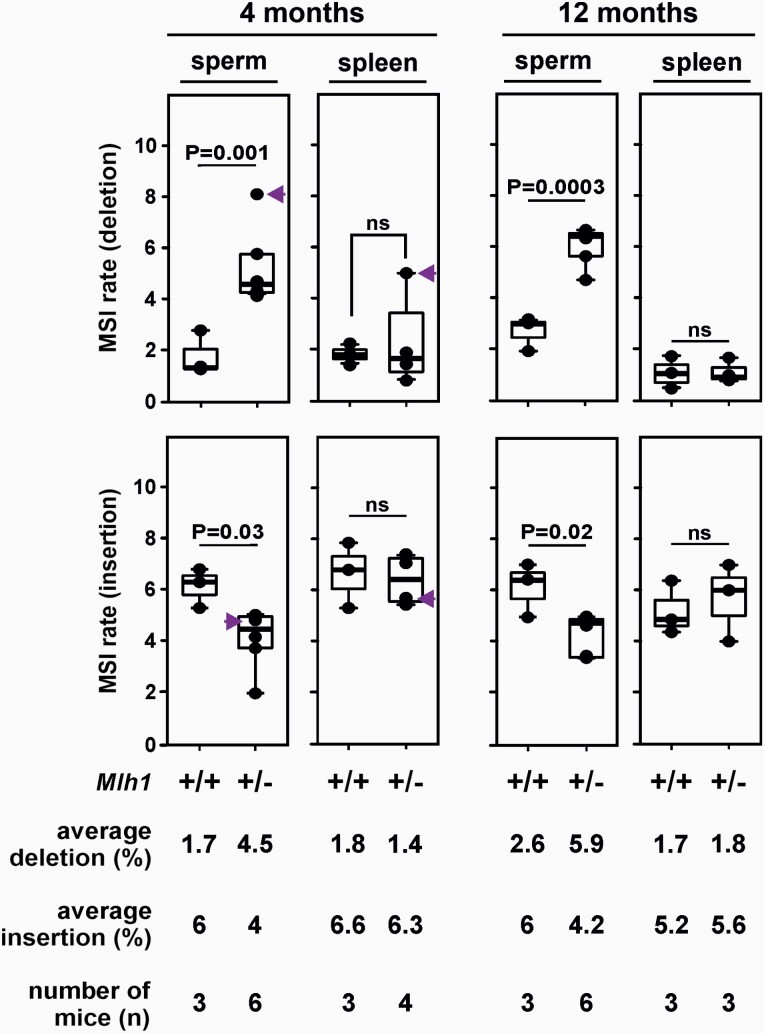
Germline versus somatic MSI. The boxplots show weighted average of MSI at mononucleotide repeats A27 and A33 in sperm and spleen of 4- and 12-month *Mlh1*^+/+^ and *Mlh1*^+/−^ mice (sperm MSI data are the same as in Figure 1C). Indicated are the *P* values for wild-type versus* Mlh1*^+/−^ MSI rate comparisons using unpaired *t*-test (abbreviation: ns, non-significant). Arrow indicates the outlier *Mlh1*^+/−^ mouse which was excluded from statistical analysis.

We also performed the SM-PCR-based MSI assay in spleen, which enabled us to compare germline versus somatic MSI for each mouse. As in sperm, the dinucleotide locus D14Mit15 was also stable in spleen (Supplementary Figure 5), and therefore, tissue-specific MSI was compared only for mononucleotide repeats. Both sperm and spleen of wild-type mice showed only baseline levels of deletions at mononucleotide microsatellites. In *Mlh1* heterozygotes, the increase in deletions was near-exclusive to sperm, the exception being spleen DNA in the outlier mouse (Figure 3). Deletions in *Mlh1*^+/−^ sperm were significantly higher than in spleen (2.5- and 3.2-fold at 4- and 12-month time points, respectively, Figure 3; *P* values = 0.0087 and 0.0052).

In wild-type mice, both sperm and spleen DNA at both time points had an insertional burden (Figure 3). The substantial decrease in insertions, seen in *Mlh1*^+/−^ sperm compared to *Mlh1*^+/+^ sperm, was not observed in *Mlh1*^+/−^ spleen (Figure 3).

## Discussion

There is emerging evidence that phenotypically normal somatic tissues from individuals with inherited MMR heterozygosity display MSI (37,38), raising the question whether germline cells of such individuals also exhibit MSI. Further, *Mlh1* promoter methylation in sperm of LS patients has been reported (21,39,40) but little is known about how MMR heterozygosity and *Mlh1* promoter methylation impact sperm MSI. We now demonstrate MSI in sperm of *Mlh1* heterozygotes, and show that *Mlh1* promoter methylation is frequent in *Mlh1*^+/−^ sperm and associates with sperm MSI. Our experimental design (i.e. a highly sensitive, single-DNA molecule-based MSI assay) allowed detection of MSI as low as 1%. All samples assayed showed <10% MSI, with a standard MSI assay [with a detection limit of 20–25% MSI (41)], this level of MSI would have been missed.

Interestingly, all *Mlh1*^+/−^ mice harbouring *Mlh1* promoter methylation in sperm also showed *Mlh1* promoter methylation in spleen. Our observation is in line with the human studies where LS and LS-like (individuals with germline MMR promoter methylation) patients are reported to have *Mlh1* promoter methylation in multiple tissues analysed, including sperm cells (21,40,42–44). We show, for the first time, that sperm *Mlh1* promoter methylation is common in *Mlh1*-heterozygous mice. Further, we demonstrate that *Mlh1* heterozygosity and promoter methylation associates with MSI in sperm but not in spleen of the same mouse. This result is perhaps not surprising, given that the likelihood of MSI increases with each round of DNA replication, and spermatogenesis involves sustained proliferation of spermatogonia (45) while splenocytes have a much lower proliferation rate (46).

We recently reported an insertional burden in a somatic tissue with fully proficient MMR (*Mlh1*^+/+^) (47), others have shown similar insertional burden in MMR-proficient mammalian cell lines (48,49). In proliferative somatic tissues, insertions tend to substantially decrease with decreasing MMR dosage (i.e. from *Mlh1*^+/+^ to *Mlh1*^+/−^ to *Mlh1*^−/−^ tissue) with barely detectable insertions in MMR-deficient tissues and in MMR-deficient tumours, while deletions show the opposite trend. Insertions or deletions at microsatellites are thought to originate from DNA polymerase slippage that results in the formation of a small loop on the newly synthesised or the template strand during DNA replication, respectively (50–52). Our observation of increase in deletions (and decrease in insertions) in *Mlh1* heterozygotes implies increased DNA polymerase slippage on the template strand and/or less efficient repair of the resulting template strand loops when MMR activity is not fully proficient. A likely explanation for the apparently lower insertion rate in *Mlh1* heterozygotes is that deletion events erase many insertions that arose during earlier cell divisions. A tug-of-war between insertions and deletions in *Mlh1* heterozygotes could mean that we are substantially underestimating the true extent of ongoing MSI with our molecular read-out. Regardless, we now demonstrate that the differential accumulation of insertions versus deletions at mononucleotides repeats, reported before in somatic tissues, bear out also in male germline cells.

Overall, the maintenance of genome stability in the germline is crucial in order to avoid passing on any *de novo* defects to offspring. We have demonstrated that MMR heterozygosity provokes elevated MSI in male gametes. Further, our study highlights the utility of *Mlh1* mice to study MMR-associated epigenetic phenotypes and MMR epimutation.

## Supplementary data

Supplementary data are available at *Mutagenesis* Online.

### Mlh1 genotyping

*Mlh1* genotyping was performed using earpieces (53). Earpieces were lysed overnight at 56°C using 100 µl of lysis buffer [10 mM Tris-HCl (pH 8.3), 50 mM KCl, 2.5 mM MgCl_2_, 0.1 mg/ml gelatin, 0.45% Tween20, 0.45% NP-40] supplemented with 20 µg proteinase K. Proteinase K inactivation was performed by boiling the lysate for 10 min, and the lysate was spun down for 1 min at 14 000 rpm. 0.5 µl of the supernatant was seeded into the genotyping PCR. PCR was performed using Platinum Green Hot Start PCR master mix (Invitrogen, Carlsbad, CA, USA) with *Mlh1* genotyping primers published by the Frederick national laboratory mouse repository (https://frederick.cancer.gov/science/technology/MouseRepository/MouseModels/Protocols.aspx?s=01XA2&g=Mlh1&p=1), namely primers M001 5′-TGTCAATAGGCTGCCCTAGG-3′, M002 5′-TGGAAGGATTGGAGCTACGG-3′, and M003 5′ TTTTCAGTGCAGCCTATGCTC-3′. The PCR program was as follows: 2 min at 94°C, 38 cycles of 30 s at 98°C, 30 s at 55°C and 45 s at 72°C, followed by final elongation of 5 min at 72°C. Primer combination M001/M002 and M001/M003 amplifies knockout and wild-type allele, respectively.

Supplementary Fig. 1. Single-molecule MSI analysis at dinucleotide repeat D14Mit15 in sperm. (**A**) Representative capillary electropherograms of most abundantly observed alleles of the D14Mit15 microsatellite: 1 repeat unit insertion (top panel), wild-type allele (middle panel) and 1 repeat unit deletion (bottom panel). Highlighted peaks are the true microsatellite peaks that were scored, smaller peaks preceding the true peak are stutter peaks. (**B**) Very few mutant alleles were observed at D14Mit15. On the *y*-axis, ‘+’ indicates insertions and ‘-’ indicates deletions. (**C**) MSI at D14Mit15. Indicated are the *P* values for wild-type and *Mlh1*^+/−^ sperm MSI rate comparisons using unpaired *t*-test (abbreviation: ns, non-significant). White dots indicate mice with *Mlh1* promoter methylation.

Supplementary Fig. 2. Schematic diagrams of MSI at A27, A33 and D14Mit15. Each repeat unit of the microsatellite is shown as a coloured rectangle, along with 10 nucleotides flanking the microsatellite locus. Wild-type alleles of A27, A33 and D14Mit15 consist of (A)_27_, (A)_33_ and (CA)_21_ repeat units, respectively. Examples of most common mutant alleles (1-repeat unit insertions and 1-repeat unit deletions) are shown above and below the wild-type allele.

Supplementary Fig. 3. Insertions at mononucleotide repeats in sperm and the *Mlh1* promoter methylation status. Each bar represents sperm from an individual mouse. ‘M’ indicates mice with *Mlh1* promoter methylation in sperm. Data are presented in order of samples in the gel image in Figure 2A. Arrow indicates the outlier *Mlh1*^+/−^ sperm sample.

Supplementary Fig. 4. *Mlh1* promoter methylation in spleen. Representative gel image of MSP for the *Mlh1* promoter. Samples from individual mice are in the same order as in gel image in Figure 2A.

Supplementary Fig. 5. Single-molecule MSI analysis at dinucleotide repeats D14Mit15 in spleen. (**A**) Allele frequency (%) of various mutants observed at D14Mit15. On the *y*-axis, ‘+’ indicates insertions and ‘-’ indicates deletions. (**B**) MSI at D14Mit15. Indicated are the *P* values for wild-type and *Mlh1*^+/−^ spleen MSI rate comparisons using unpaired *t*-test (abbreviation: ns, non-significant). White dots indicate mice with *Mlh1* promoter methylation.

## Supplementary Material

geab010_suppl_Supplementary_Material
