# Supplementary material for: Mlh1 heterozygosity and promoter methylation associates with microsatellite instability in mouse sperm
Source: Mutagenesis. 2021 Mar 19;36(3):237–44. doi: 10.1093/mutage/geab010 (PMC8262379; doi:10.1093/mutage/geab010)
Supplement: geab010_suppl_Supplementary_Material [file geab010_suppl_supplementary_material.docx]

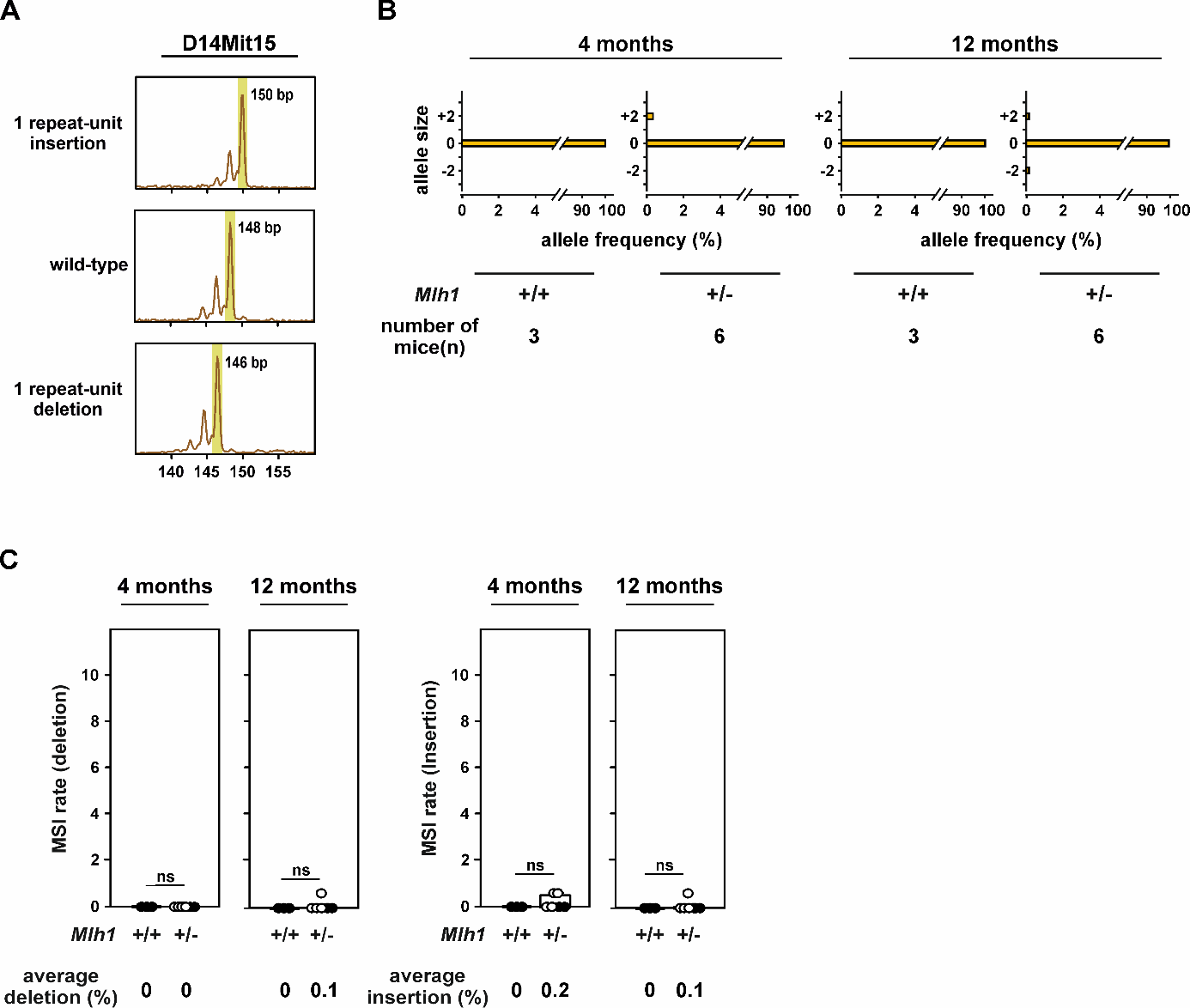


**Supplementary fig. 1. Single-molecule MSI analysis at dinucleotide repeat D14Mit15 in sperm.**

**
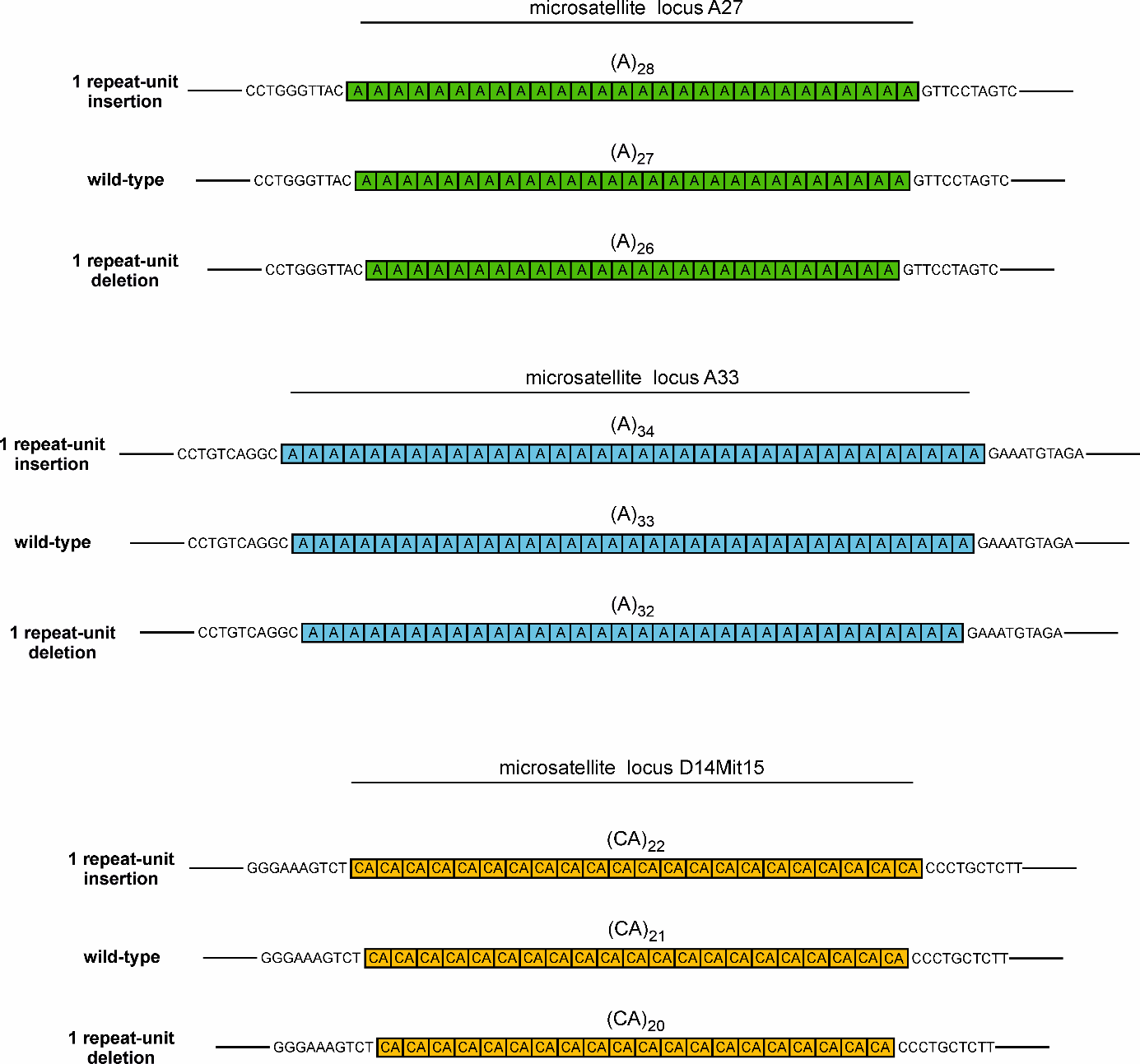
**

**Supplementary fig. 2**. **Schematic diagrams of MSI at A27, A33 and D14Mit15.**


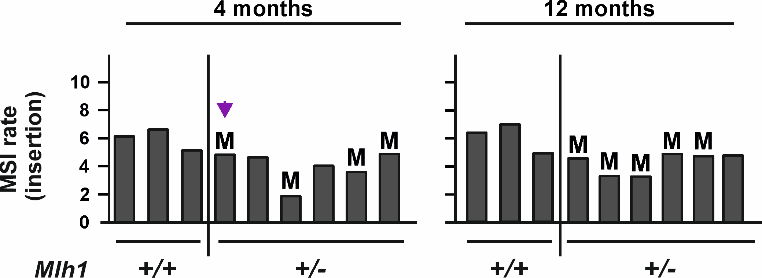


**Supplementary fig. 3. Insertions at mononucleotide repeats in sperm and the *Mlh1* promoter methylation status.**

**
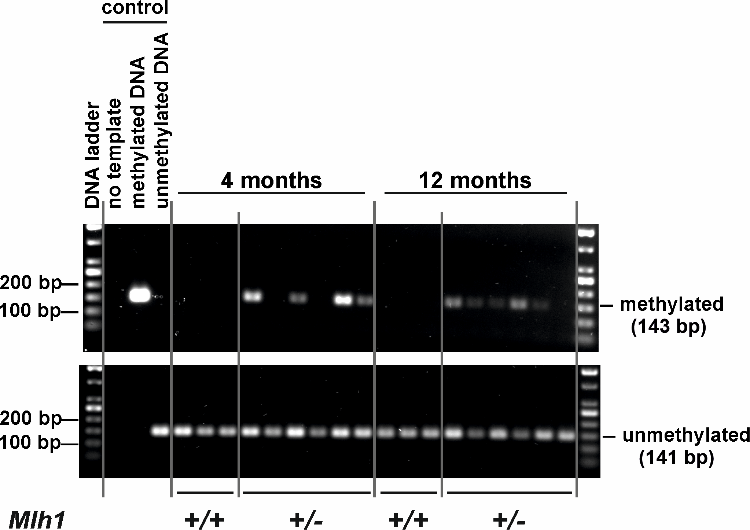
**

**Supplementary fig. 4. *Mlh1* promoter methylation in spleen.**

**
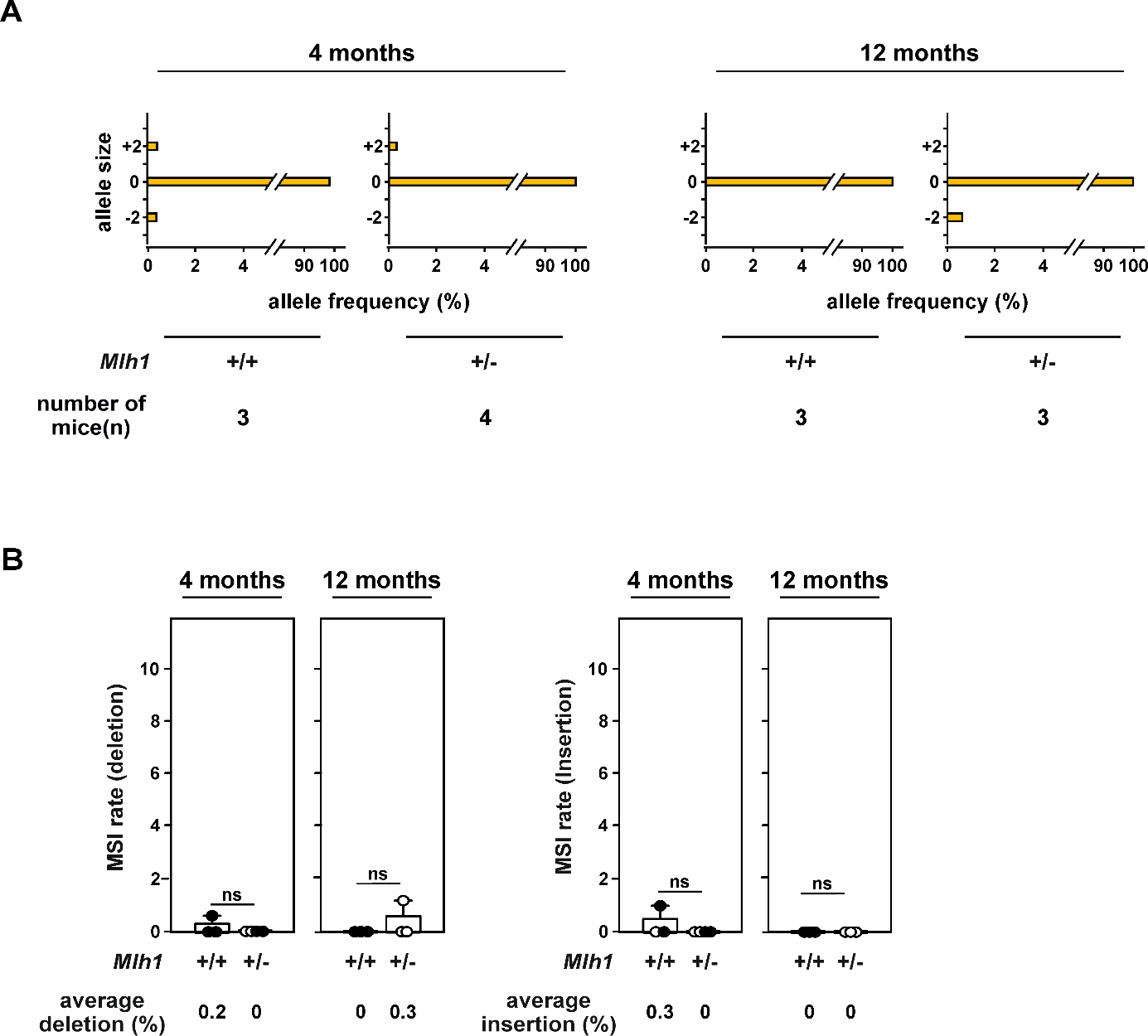
**

**Supplementary fig. 5. Single-molecule MSI analysis at dinucleotide repeat D14Mit15 in spleen.**
